# Effects of Pu-erh and Dian Hong tea polyphenols on the gut-liver axis in mice

**DOI:** 10.1186/s13568-023-01565-4

**Published:** 2023-06-02

**Authors:** Ning Wang, Chaohua Lan, Muhammad Aamer Mehmood, Manli He, Xiongjun Xiao, Linman Li, Dalong Liao, Kewei Xu, Shan Mo, Puyu Zhang, Xiaoli Zhou, Baoxiang Gu, Hui Zhu, Tao Wu

**Affiliations:** 1 College of Bioengineering, Sichuan University of Science and Engineering, Zigong, 643000 China; 2 School of Food and Biological Engineering, Xihua University, Chengdu, 610039 China; 3 Laboratory Animal Center, Southwest Medical University, Luzhou, Sichuan China; 4 Department of Bioinformatics and Biotechnology, Government College University Faisalabad, Faisalabad, Pakistan; 5 Luzhou Laojiao Co. Ltd, Luzhou, China; 6 College of Horticulture, Hunan Agricultural University, Changsha, China

**Keywords:** Tea polyphenols, Intestinal microbiota, Lipid metabolism, Antioxidative

## Abstract

Tea polyphenols (TP) are the most biologically active components in tea, with antioxidant, antiobesity, and antitumor properties, as well as the ability to modulate the composition and function of intestinal microbiota. This experimental study evaluated the chemical constituents of polyphenols in Pu-erh (PTP) and Dian Hong tea (DHTP). It also investigated the co-regulatory effects of PTP and DHTP on intestinal flora and liver tissues in mice using 16 S rRNA gene and transcriptome sequencing. The results revealed that DHT had higher concentrations of EGC (epigallocatechin), C (catechin), EC (epicatechin), and EGCG (epigallocatechin gallate). In contrast, PT had higher concentrations of GA (gallic acid), ECG (epicatechin-3-gallate), TF (theaflavin), and TB (theabrownin). PTP and DHTP consumption significantly reduced the rates of weight gain in mice. Microbial community diversity was significantly higher in PTP and DHTP-treated mice than in the control group. Notably, beneficial microbes such as *Lactobacillus* increased significantly in PTP-treated mice, whereas *Lachnospiraceae* increased significantly in DHTP-treated mice. Both PTP and DHTP improved the activity of the antioxidant enzymes (SOD) and total antioxidant capacity (T-AOC) in the liver. The transcriptome analysis revealed that the beneficial effects of PTP and DHTP were due to changes in various metabolic pathways, the majority of which were related to antioxidant and lipid metabolism. This study discovered that PTP and DHTP had beneficial effects in mice via the gut-liver axis.

**Supplementary Information:**

The online version contains supplementary material available at 10.1186/s13568-023-01565-4.

## Key points

- Compare the chemical components of Pu-erh and Dian Hong tea.
- Polyphenols from Pu-erh and Dian Hong tea can regulate different intestinal microorganisms.
- Polyphenols from Pu-erh and Dian Hong tea can regulate different metabolic pathways.

**Supplementary Information:**

The online version contains supplementary material available at 10.1186/s13568-023-01565-4.

## Introduction

The intestinal microbiota is critical to human health because it is a complex micro-ecological environment with its own metabolic mechanism (Cani 2018). Recent studies indicated the involvement of intestinal microbiota in various human physiological processes, including regulating metabolism and immunity. Chronic diseases such as obesity, inflammatory bowel disease, diabetes mellitus, metabolic syndrome, and nonalcoholic fatty liver disease are closely associated with disorders of the intestinal microbiota (Aron-Wisnewsky et al. 2021; Lynch and Pedersen 2016; Ma et al. 2019). The intestine and liver are closely connected through anatomy and biological functions. They form the so-called “gut-liver axis,“ by which the intestinal bacteria communicate with the liver through portal circulation (Albillos et al. 2020). Various metabolites produced by the bacteria are absorbed by the small intestine and then transported directly to the liver through the portal vein, either as energy or as signaling and regulatory molecules (Albillos et al. 2020; Milosevic et al. 2019). Endogenous or exogenous environmental factors can alter the intestinal microbial population. For example, dietary interventions can regulate gut microbiota. Moreover, diet presents outstanding advantages such as low side effects and high sustainability.

Tea is one of the world’s most popular beverages (Brody 2019). Black tea accounts for 70–80% of global tea production (Tanaka and Matsuo 2020). Yunnan, China, is one of the most important black tea-producing regions in the world. Pu-erh (PT) and Dian Hong tea (DHT) are well-known tea brands made from *Camellia sinensis* trees grown in Yunnan, China.

PT is a dark tea made through a lengthy pile-fermentation process. DHT, on the other hand, is produced through fermentation using endogenous enzymes found in tea leaves, such as polyphenol oxidase (Gong et al. 2012; Li et al. 2020). Both PT and DHT are particularly rich in pigments, such as theabrownins, thearubigins, and theaflavins, which are generated by the oxidation of tea phenolics (LI 2009). Theoretically, tea pigments do not easily penetrate the intestinal barrier and are present in low concentrations in the serum. However, some reports indicate that obese mice on a high-fat diet derive immediate health benefits from consuming green and black tea (Wang et al. 2017). Therefore, tea may have various prebiotic effects by regulating intestinal microbes. However, there may be differences in the way and mechanism by which different teas regulate probiotics, and there are currently few comparative studies on different teas.

Pu-erh tea Polyphenols (PTP) and Dianhong Tea Polyphenols (DHTP) were isolated and evaluated from the same tea raw material at different fermentation levels in this study. The effects of the polyphenols in both teas were investigated on the body weight, serum, liver antioxidant capacity, and intestinal microbiota of C57BL/6j mice. The aim was to elucidate the regulatory pathways and molecular mechanisms responsible for the potentially beneficial effects of Pu-erh and Dian Hong tea polyphenols.

## Materials and methods

### Preparation of the tea extracts

The Dian Hong tea and Pu-erh tea samples prepared from the same raw materials were provided by Sichuan Jixiang Tea Co. Ltd. The Dianhong tea and Pu-erh tea samples (10 g each) were first crushed and passed through an 80-mesh sieve. The powder was decocted twice in 50-fold boiling water for 10 min. The supernatant was collected by centrifugation at 5000 rpm for 10 min and concentrated under reduced pressure using a rotary evaporator. The Dian Hong tea extract (RTe) and Pu-erh tea extract (PTe) were lyophilized under 10 Pa at −55 °C and stored at 4 °C for further exploration.

### Determination of the primary compounds in Pu-erh and Dian Hong tea

For the quantitative detection of the major constituents in tea samples, an LC-30 UHPLC system (Shimadzu, Japan) equipped with a reversed-phase C18 column (150 mm × 2.1 mm, 3.5 μm, Agilent Eclipse XDB) was used. The gradient conditions were performed exactly as described previously (Xu et al. 2019).

#### Extraction of tea polyphenols

Pu-erh and Dianhong tea were extracted by heating water 3 times and combining the extracts. Tea extract is adsorbed by a macromolecule adsorbent and then eluted by a 90% ethanol solution, allowing the tea polyphenols adsorbed on the adsorbent to be desorbed in ethanol and recovered via vacuum distillation. To obtain tea polyphenols, the concentrated solution is vacuum dried.

### Animal design and ethical statement

SPF Biotechnology Co. Ltd (Beijing, China) provided the C57BL/6j mice (15–18 g, male), which were kept in light/dark conditions at 22 °C for 12 h/12 h, with unlimited access to water and a standard rodent diet. After one week of acclimation, 24 mice were randomly assigned to one of three experimental groups: control (H), Dian Hong tea (R), and Pu-erh tea (P), each with 8 mice. The mice in the R and P groups were orally treated with 100 mg/kg body weight of the DHTP and PTP, respectively. The mice in the H groups were given ddH_2_O of the same volume. The body weight was monitored and recorded every two days throughout the experimental period.

The mice were sacrificed on day 15, and the liver tissues were collected and frozen overnight in liquid nitrogen for RNA extraction. Blood samples were collected, refrigerated overnight at 4 °C, and then centrifuged at 10,000 rpm for 10 min. The serum was collected to detect the antioxidant capacity. The cecal contents were dissected and stored at −80 °C.

After obtaining proper approval (swmu20220137), all experiments were carried out in accordance with the Southwest Medical University Guide for Care and Use.

### DNA extraction and sequencing

The CTAB/SDS method was used to extract total DNA from all cecal samples (75 mg each). The DNA quality was assessed on a 1% agarose gel electrophoresis, and the quantity was estimated on the NanoDrop Spectrophotometer (ND-1000, Thermo Fischer Scientific, USA). The DNA was used as the template to amplify the V4 hypervariable region of the 16 S rRNA gene and sequenced at Novogene Bioinformatics Technology Co. Ltd. (Beijing, China).

### Bioinformatics and statistical analyses

The single-end reads were cleaned by removing the primer sequences and subjected to quality filtration following the recommended quality control parameters of the Cutadapt V1.9.1 tool. The chimera sequences were removed using the SILVA reference database and UCHIME algorithm (Edgar et al. 2011). The cleaned reads were analyzed by UPARSE v7.0.1001. Sequences showing ≥ 97% similarity were grouped into the same OTUs. The SILVA Database and Mothur algorithm were employed to assign taxonomy to the representative sequences of each OTU. Finally, multiple sequence alignments were performed using the MUSCLE v3.8.31 software. Alpha diversity and Jackknifed beta diversity analysis were performed.

#### Determination of the total antioxidant (T-AOC) capacity in mice

Liver tissue (0.1 g) was added into 1.0 mL of precooled T-AOC extraction solution and homogenized at 4 °C for 1 min. The supernatant was collected after centrifugation at 10,000 rpm for 5 min. The reaction solutions were mixed according to the instructions of the Total Antioxidant Capacity (T-AOC) Colorimetric Assay kit (Solarbio Science & Technology Co. Ltd. Beijing, China), and the absorbance was measured at 595 nm. The T-AOC values of the serum and liver tissue samples were calculated using the standard curve (Y = 10.829X + 0.0146, R^2^ = 0.9995). The experiments were repeated thrice.

### Determination of the superoxide dismutase (SOD) activity in mice

The pretreated liver homogenate and serum samples were mixed with the reaction solution following the instructions of the Superoxide Dismutase (SOD) Colorimetric Activity Kit (Solarbio Science & Technology Co. Ltd. Beijing, China) and then incubated at room temperature for 30 min. After measuring the absorbance at 560 nm, the SOD activity of the serum and liver tissue was calculated.

### RNA isolation to construct a library

Three representative liver samples were selected from each group (LH, LR, LP), and the total RNA was extracted using the TRIzol reagent following the manufacturer’s instructions (Invitrogen, Carlsbad, USA). After determining the RNA quality and quantity, the libraries were constructed and sequenced on an Illumina HiSeq X Ten platform (Illumina Inc. San Diego, USA). Further, 150 bp paired-end reads were obtained.

### RNA-Seq analyses

Two groups/conditions (in duplicate) were subjected to differential expression analyses using the DESeq2 R package (1.16.1). Gene Ontology (GO) analysis of the differentially expressed genes (DEGs) was performed using the clusterProfiler R package, and the gene length bias was corrected. GO terms with corrected *P < 0.05* were considered enriched by DEGs. The statistical enrichment of the DEGs in the KEGG pathways was tested through the clusterProfiler R package.

### Quantitative real-time PCR

qRT-PCR was performed to validate the DEGs obtained from the RNA-Seq results. From these genes, six antioxidant-related genes (COX-2, iNOS, IκB-α, Cu/Zn-SOD, Mn-SOD, GSH-Px) and six lipid metabolism-related genes (PPAR-α, LDLR, CPT-1a, C/EBP-α, FAS, SREBP-1c) were selected.

The high-quality RNA extracted from the liver tissues was used as the template to synthesize cDNA. The Real-Time quantitative PCR (RT-qPCR) reaction was executed to detect the change in the expression of genes using the 2^−ΔΔCt^ method. The β-actin gene was the reference, and normal mice were the controls (Additional file 1: Table S1).

### Statistical analyses

All experiments were repeated thrice. Data were represented as mean ± SD values. One-Way ANOVA with Tukey’s post hoc test was employed using GraphPad Prism 6.0 (San Diego, USA). Data with p-values < 0.05 were considered significant.

## Results

### Characterization of the active ingredients in PT and DHT extracts

The total phenolic contents estimated by the Folin-Ciocalteu method were 11.6 g GAE (gallic acid equivalent)/100 g and 9.12 g GAE/100 g in DHT and PT, respectively. Furthermore, UHPLC analysis identified the significant ingredients in the two tea extracts. Higher concentrations of EGC (epigallocatechin), C (catechin), EC (epicatechin) and EGCG (epigallocatechin gallate) were present in DHT than in PT. In contrast, higher levels of GA (gallic acid), ECG (epicatechin-3-gallate), TF (theaflavin), and TB (theabrownin) were found in PT (Table 1).

**Table 1:** Comparison of compound contents of DBT and PT (mg/g)

Compounds	DBT	PT
GA	2.16 ± 0.11a	11.63 ± 0.14a
EGC	31.38 ± 0.42a	3.27 ± 0.32a
C	1.52 ± 0.15	1.18 ± 0.12
EC	2.34 ± 0.16a	1.46 ± 0.10a
EGCG	7.63 ± 0.4a	1.65 ± 0.62a
ECG	15.19 ± 0.7a	35.2 ± 0.74a
TB	2.31 ± 0.12a	4.39 ± 0.05a
TF	0.21 ± 0.01a	1.34 ± 0.01a

### Sequencing metadata and microbial diversity in the gastrointestinal tracts of the mice

After removing low-quality reads and chimeras, 19,23,284 high-quality 16 S rRNA gene sequences (V4 region: 533–786 bp) with no chimeras were obtained. The average was 80136.83 ± 86.63 sequences per sample, ranging from 80,034 to 80,344. The average length of these sequences was 411 bp, and they belonged to 1703 operational taxonomic units (OTUs) based on 97% similarity. Each sample had an average of 567.67 ± 7.09 OTUs. After calculation, the effective data accounted for 98%. The average Q20 was 80.67, and the average GC ratio was 52.79% (Additional file 1: Table S2).

**Fig. 1:**
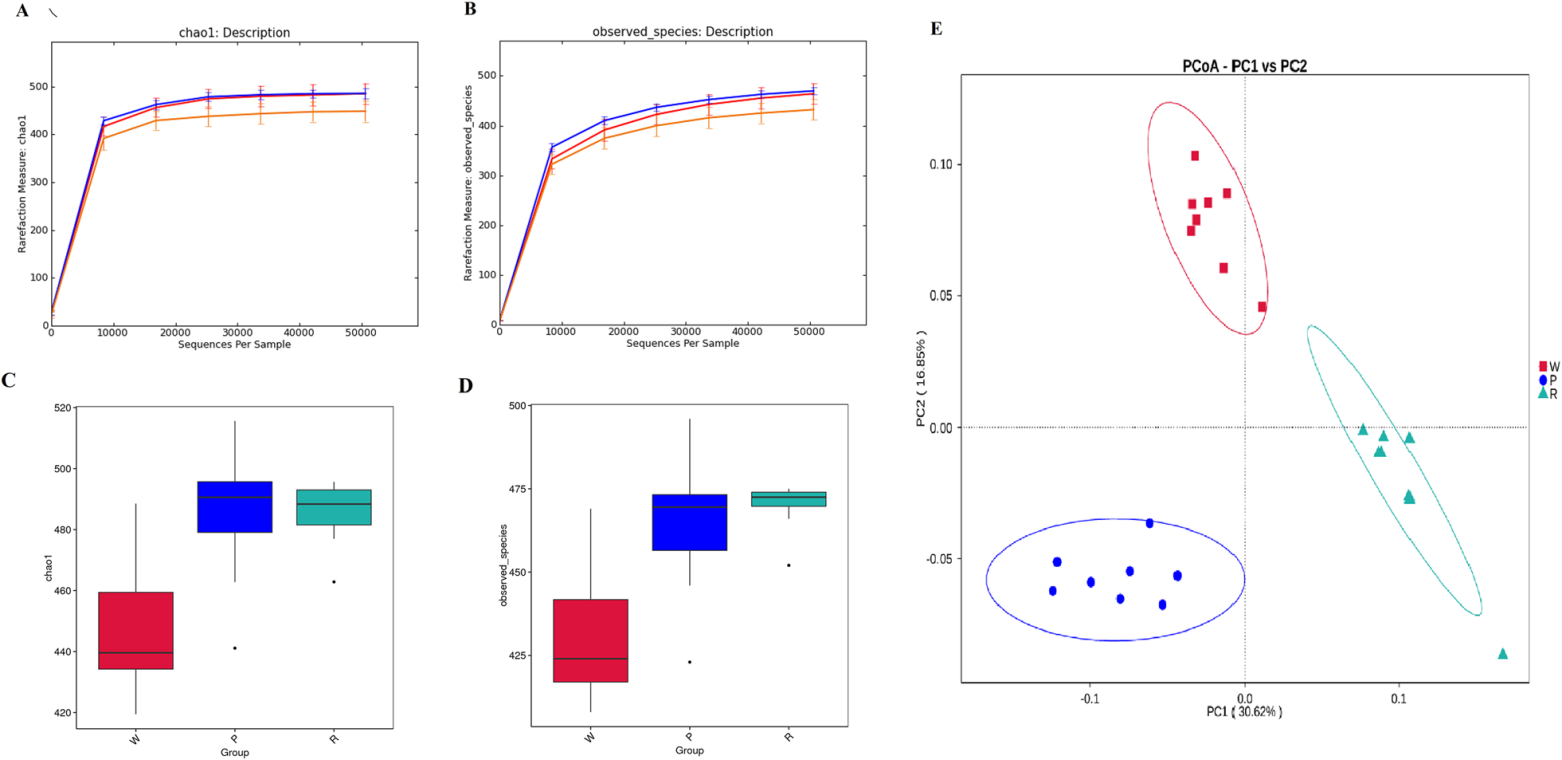
Microbial diversity in the gastrointestinal tracts of the mice species diversity curve, **A** Chao1 index, **B** Observed species; Statistical map of species significant differences between groups, **C** Chao1 index, **D** Observed species; **E** Based on unweighted_unifrac level of PCOA analysis result graph, Each point in the figure represents a sample, and samples from the same group are represented by the same color

The microbial community richness (alpha diversity) was measured using the Chao1 index, and species were identified. The indices did not differ significantly between the P and R groups. However, the Chao1 and Observed species were notably different in the bacterial community structure of P vs. H and R vs. H. Microbial community diversity was significantly higher in mice treated with PTP and DHTP than in the control group. (Fig. 1A, B, C and D).

The beta diversity representing the differences among the H, P, and R mice was examined using weighted and unweighted UniFrac distances. PCoA (Principal coordinates analysis) visualized the distances. The mice treated with ddH_2_O, Dian Hong tea polyphenols, and Pu-erh polyphenols harbored distinct microbial taxa. The microbial communities in the mice belonging to the H, P, and R groups clustered together and separated along the principal coordinate axis based on membership (Fig. 1E). ANOSIM (Analysis of similarities) (P < 0.01) identified significant differences in the community memberships between the different groups. Thus, distinct microbial community structures existed among the mice treated with different tea polyphenols.

### Differences in the microbial communities of mice treated with ddH_2_O_2_, Dian Hong tea polyphenols, and Pu-erh tea polyphenols

**Fig. 2:**
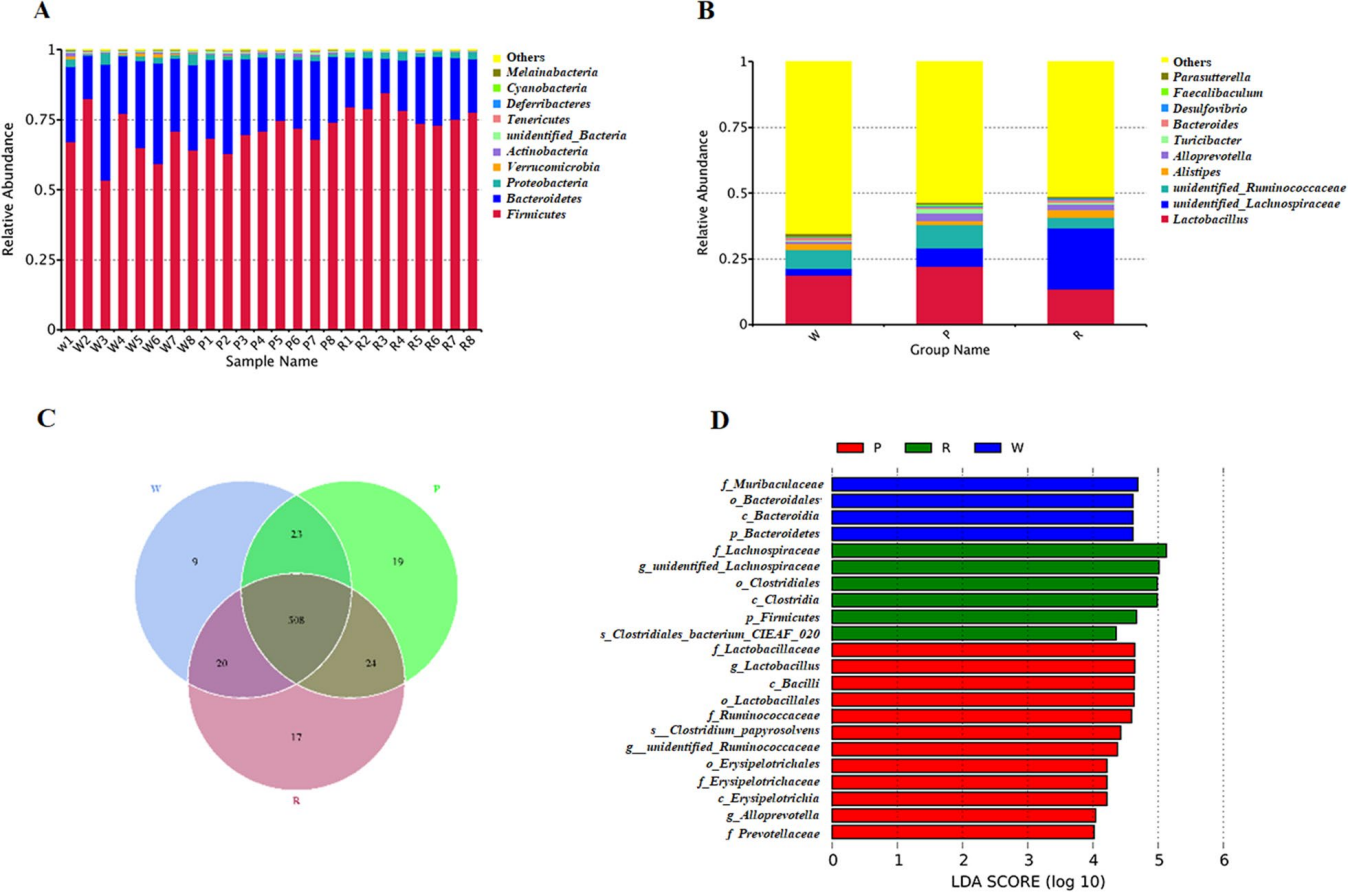
Differences in the microbial communities of mice treated with ddH_2_O, Dianhong black tea, and Pu-erh tea polyphenols. **A** Histogram of relative abundance of species at the gate level; **B** Column Chart of Relative Species Abundance at Genus Level; **C** Venn Graph; **D** LDA value distribution histogram

The specific taxonomic groups of species (e.g., kingdom, phylum, class, order, family, genus, and species) were identified. Two groups of bacteria, the *Firmicutes* and *Bacteroidetes*, dominated the intestinal microbiota of these mice, accounting for more than 97% of reads. The relative abundance of these two predominant microbes (phylum level) differed between the mice groups. The mice in the H, P, and R groups contained 67.48%, 70.11%, and 77.65% of *Firmicutes*, and 28.41%, 26.65%, and 19.44% of *Bacteroidete*s (Fig. 2A). *Lactobacillus, Lachnospiraceae, Ruminococcaceae, Alistipes, Alloprevotella, Turicibacter, Bacteroides, Desulfovibrio, Faecalibaculum*, and *Parasutterella* were dominant at the genus level (Fig. 2B).

The Venn diagrams consisted of three groups and 620 OTUs via clustering. The cluster numbers of OTUs in the R, H, and P groups were 569, 560, and 574, respectively. Of these, the R and H groups shared 528 OTUs in the overlapping portion, the P and H groups shared 531 OTUs, and the P and R groups shared 532 OTUs. Moreover, 19 species were unique to the P group, nine to the H group, and 17 to the R group (Fig. 2C).

The LEfSe (Linear discriminant analysis Effect Size) tool focuses on the significant differences and biological relevance. It identified specific genera that were differentially distributed among the H, P, and R groups. Furthermore, 22 genera were differentially represented among the three groups, 12 genera were more abundant in the P group (e.g., *Lactobacillaceae*, *Lactobacillus* spp. *Bacilli*, *Lactobacillales*, *Ruminococcaceae*, *Clostridium papyrosolvens*, *Ruminococcaceae* spp. *Erysipelotrichales*, *Erysipelotrichia*, *Alloprevotella* spp. and *Prevotellaceae*). Further, six genera were more abundant in the R group (e.g., *Lachnospiraceae*, *unidentified Lachnospiraceae* spp. *Clostridiales*, *Clostridia*, *Firmicutes*, and *Clostridiales bacterium CIEAF 020*) (Fig. 2D).

### Effect of tea polyphenols on the body weight of mice

**Fig. 3:**
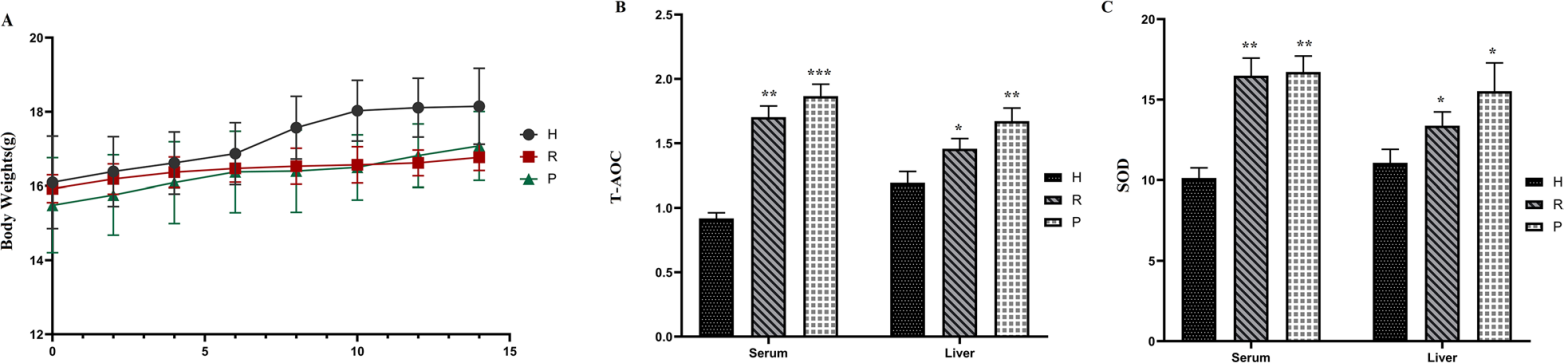
Effects of Dian Hong tea and Pu-erh tea polyphenols on the body parameters of mice. **A** Body weights (g). **B** T-AOC activity in serum and liver. Serum: U/mL, Liver: U/g. **C** SOD activity in serum and liver. Serum: U/mL, Liver: U/g. *p < 0.05, **p < 0.01, ***p < 0.005 vs. the H group

There was no significant difference in the average body weight of mice in each group at the beginning. During the experimental period, no significant difference was seen in the food and water intake among the mice in different groups. However, treatment with DHTP and PTP for 14 days caused a much slower weight gain in the R and P groups than in the H group. The rates of weight gain in the R and P groups were 5.27% and 10.29%, respectively, while it was 12.74% in the H group (Fig. 3A).

### Analysis of antioxidant capacity of serum and liver tissue in mice

To assess the antioxidant capacity of the two tea polyphenols, we measured the activities of T-AOC and SOD in mouse serum and liver. T-AOC and SOD activities in serum and liver tissue of groups R and P were significantly higher than those in group H. (Fig. 3B, C). T-AOC activity in serum samples was 1.70 ± 0.09 U/mL, 1.87 ± 0.09 U/mL, and 0.92 ± 0.04 U/mL, respectively, while SOD activity was 16.48 ± 1.10 U/mL, 16.72 ± 0.99 U/mL, and 10.12 ± 0.63 U/mL, respectively. T-AOC activity in the liver tissue was 1.46 ± 0.08 U/g, 1.67 ± 0.10 U/g, and 1.19 ± 0.07 U/g, respectively, and SOD activity was 13.38 ± 0.85 U/g, 15.52 ± 1.76 U/g, and 11.07 ± 0.84 U/g.

### Transcriptome data analysis

The liver transcriptomic analysis was performed by sequencing the RNA samples from three liver tissues in each group. A total of 1,268,484,156 raw sequencing reads were generated, and 1,217,618,868 clean reads remained after filtering (Additional file 1: Table S3).

**Fig. 4:**
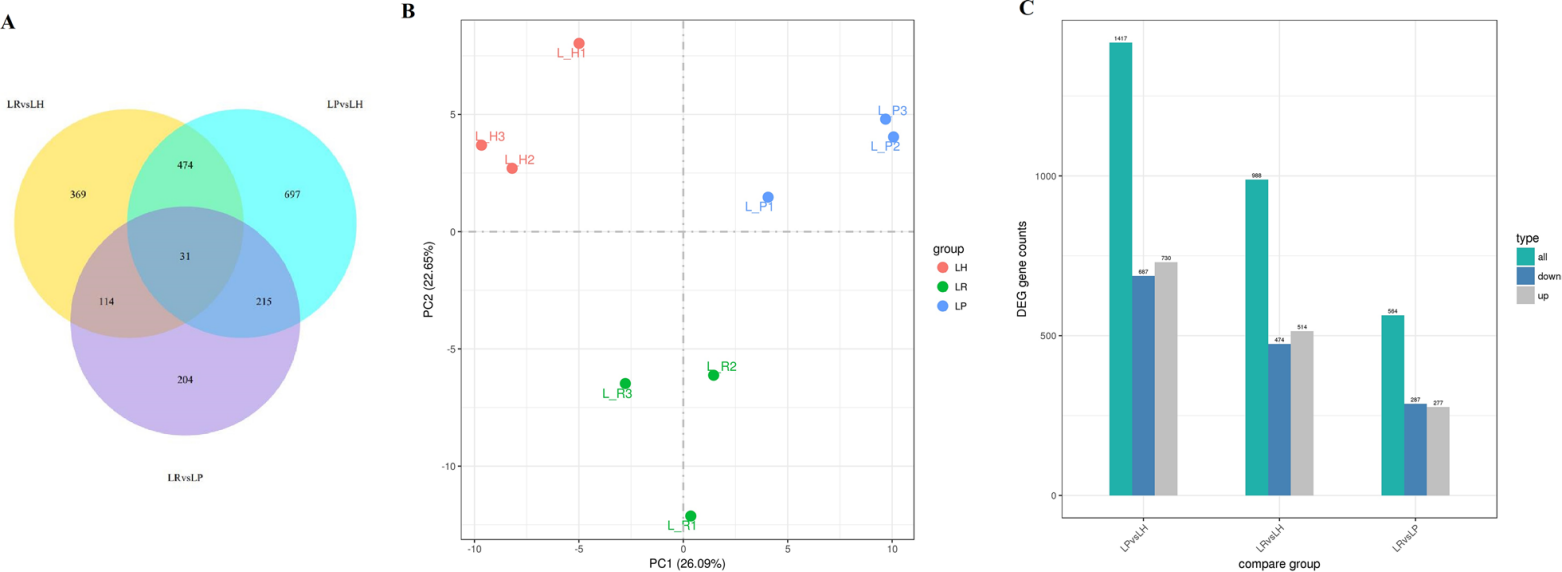
Transcriptome analysis of mice liver. **A **Venn diagram of coexpression. **B** Results of the Principal component analysis. The abscissa is the first principal component, and the ordinate is the second principal component. **C** Statistical histogram of the number of differentially compared genes in combination. Grey and blue represent upregulated and downregulated differential genes, respectively. The numbers on the bars represent the number of differential genes. *LH* Liver tissue samples from the H group of mice, *LP* Liver tissue samples from the P group of mice, *LR* Liver tissue samples from the R group of mice

Principal Component analysis (PCA) was performed on the gene expression values (FPKM) of all samples to evaluate the difference between groups and the duplication of samples within groups. The samples between LH, LR, and LP groups were dispersed, but the samples within groups were clustered together (Fig. 4B).

### Identification of differentially expressed genes

The gene expression levels of LH, LR, and LP were quantified and compared. The differentially expressed genes (DEGs) were obtained using the criteria: log2 (Fold Change) > 0 and padj < 0.05. A total of 988, 1416, and 564 DEGs were identified in the LR vs. LH, LP vs. LH, and LR vs. LP groups, respectively. Among these DEGs, 369, 697, and 204 DEGs were uniquely expressed in LR vs. LH, LP vs. LH, and LR vs. LP groups, respectively. Moreover, 31 DEGs were commonly expressed in all groups (Fig. 4A).

Significantly upregulated or downregulated genes were identified by DEGseq. Compared with the LH group, the LP group exhibited 1417 DEGs (730 upregulated and 687 downregulated genes), and the LR group showed 988 DEGs (514 upregulated and 474 downregulated genes). Further, the LR group showed 564 DEGs (277 upregulated and 287 downregulated genes) when compared with the LP group (Fig. 4C).

### GO analysis for DEGs

**Fig. 5:**
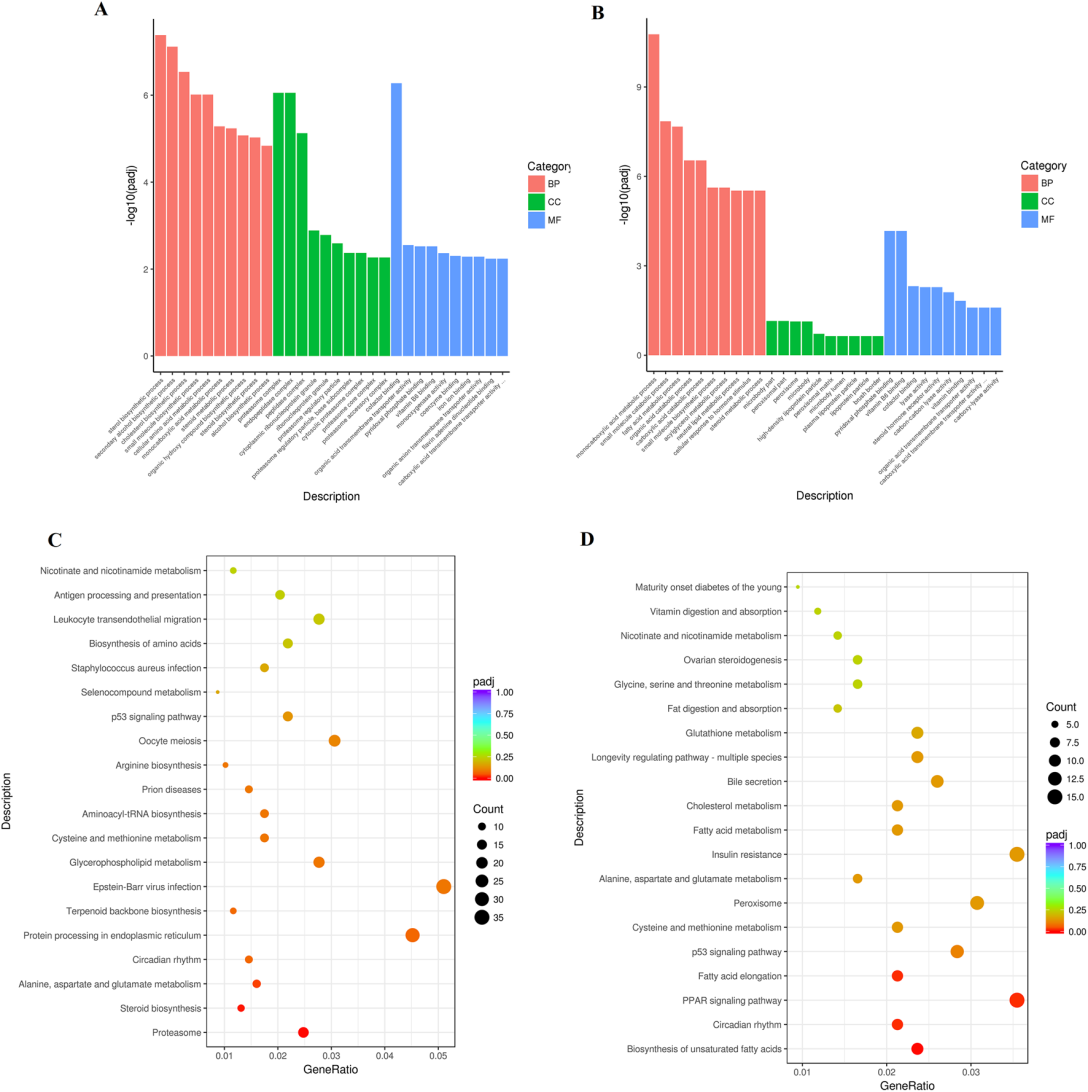
GO (Gene Ontology) and KEGG (Kyoto Encyclopedia of Genes and Genomes) pathway enrichment analyses. **A** LP vs. LH. **B** LR vs. LH. The abscissa is the ratio of the number of differential genes on the GO Term to the total number of differential genes, and the ordinate is the GO Term. *LH* Liver tissue samples from the H group of mice, *LP* Liver tissue samples from the P group of mice *LR* Liver tissue samples from the R group of mice. The abscissa is the KEGG pathway, and the ordinate is the significance level of pathway enrichment. *LH* Liver tissue samples from the H group of mice, *LP* Liver tissue samples from the P group of mice, *LR* Liver tissue samples from the R group of mice. **C** LP vs. LH. **D** LR vs. LH.

The top 30 significant GO terms for DEGs involved between LR vs. LH and LP vs. LH groups are depicted. The DEGs were mainly enriched in the sterol biosynthetic process, fatty acid metabolic process, monocarboxylic acid metabolic process, secondary alcohol biosynthetic process, proteasome complex, endopeptidase complex, oxidoreductase activity, cofactor binding, vitamin B6 binding, etc. Most genes were involved in the fatty acid metabolic process and antioxidant activity (Fig. 5A, B).

### Pathways responsive to Dianhong and Pu-erh tea polyphenols treatment

The DEGs identified between the LR vs. LH group were associated with the biosynthesis of unsaturated fatty acids, PPAR signaling pathway, p53 signaling pathway, peroxisomes, fatty acid metabolism, cholesterol metabolism, etc. (Fig. 5D). The DEGs between the LP vs. LH groups were related to proteasomes, steroid biosynthesis, protein processing in the endoplasmic reticulum, glycerophospholipid metabolism, p53 signaling pathway, etc. (Fig. 5C). The peroxisome and p53 signaling pathways were noteworthily associated with the LP and LR regulatory pathways. Most pathways regulated by PTP and DHTP were related to lipid metabolism and antioxidant action.

### Expression of genes involved in lipid metabolism and the antioxidation pathway

**Fig. 6:**
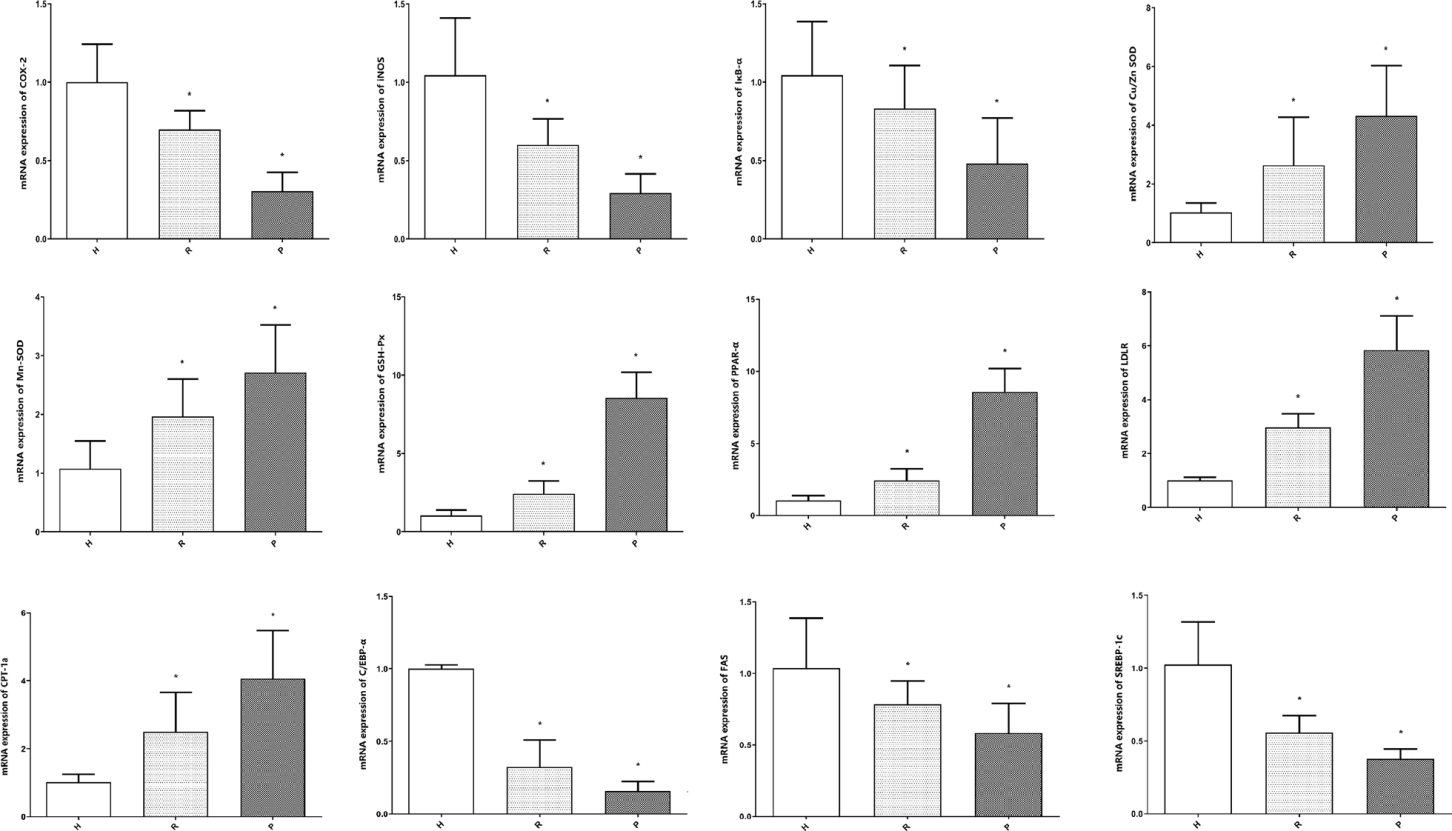
The relative mRNA expression levels of genes related to lipid metabolism and antioxidants in the liver. *p < 0.05 vs. the H group

The relative mRNA expression of six genes involved in the antioxidation signaling pathway (COX-2, iNOS, IκB-α, Cu/Zn-SOD, Mn-SOD, GSH-Px) and six genes associated with the lipid metabolism signaling pathway (PPAR-α, LDLR, CPT-1a, C/EBP-α, FAS, SREBP-1c) in the liver tissues was measured (Fig. 6).

After treatment with Dian Hong tea polyphenols, the expression of the COX-2, iNOS, and IκB-α genes in the liver was significantly reduced to 0.70-, 0.60-, 0.83-fold, while the expression of the Cu/Zn-SOD, Mn-SOD, GSH-Px genes was significantly increased to 2.64-, 1.97-, 2.43-fold compared to the mice in the LH group (P < 0.01). In the Pu-erh tea polyphenols treated mice, the mRNA expression of the COX-2, iNOS, and IκB-α genes was significantly downregulated to 0.30-, 0.29-, 0.48-fold. And the expression of Cu/Zn-SOD, Mn-SOD, and GSH-Px genes was upregulated to 4.32-, 2.71-, and 8.56-fold. In addition, higher expression of PPAR-α (2.43- and 8.56-fold), LDLR (2.96- and 5.84-fold), and CPT-1a (2.49- and 4.06-fold) was observed in these two groups. Further, lower expression of C/EBP-α (0.32- and 0.16-fold), FAS (0.78- and 0.58-fold), and SREBP-1c (0.56- and 0.38-fold) was recorded.

## Discussion

Tea polyphenols are the primary active substances in tea. They have been shown to have a variety of biological activities, including the ability to modulate the intestinal microbiota and protect the liver (Wang et al. 2022; Zhang et al. 2021). Due to different fermentation stages, the two types of tea made from the same raw material in this study have different polyphenol forms and components. The TF and TB of PTP were higher than those of DHTP, while the C of DHTP was higher. The 16 S rRNA gene sequence analysis was used to detect the intestinal microbiota of mice treated with DHTP and PTP to further investigate the biological activities of these two polyphenols with different components. PTP and DHTP had significant differences in the regulation of intestinal microbiota, according to the findings. The relative abundance of *Lactobacillus* increased significantly in mice given PTP orally, whereas *Lachnospiraceae* were abundant in mice given DHTP. *Lactobacillus* is a probiotic that is commonly used in liver protection because it improves obesity and hyperlipidemia, reduces oxidative damage and inflammation, and prevents liver damage and hepatitis, among other things (Hsieh et al. 2021; Wu et al. 2021). The *Lachnospiraceae* raise steady-state plasma levels of high-antioxidant molecules, such as hydrogen sulfide and cysteine persulfide (CysSSH), in the host (Uchiyama et al. 2022). Furthermore, producing butyrate can effectively improve diet-induced obesity, maintain the integrity of the intestinal barrier, and inhibit inflammation (Hamer et al. 2008; Hu et al. 2019; Ma et al. 2020). To summarize, PTP and DHTP were discovered to regulate various intestinal microorganisms and increase the relative abundance of beneficial bacteria that were closely related to antioxidation and lipid metabolism regulation.

Because the portal vein delivers approximately 70% of the blood to the liver, the composition and function of gut microbes can have a significant impact on liver function, particularly through the delivery of microbial-derived metabolites. (Minemura and Shimizu 2015). Because of its metabolic activities, the liver is particularly vulnerable to oxidative stress as the central organ of the lipid metabolism pathway (Cichoż-Lach and Michalak 2014). In the livers of mice treated with PTP and DHTP, antioxidant enzyme (SOD) activity and total antioxidant capacity (T-AOC) were increased. These two tea polyphenols also slowed mouse growth (weight). The expression of six vital antioxidant genes (COX-2, iNOS, IκB-α, Cu/Zn-SOD, Mn-SOD, GSH-Px) in the liver tissue were examined through qPCR to identify their role in the mice treated with the two tea polyphenols. In the current study, the gene expression of Cu/Zn-SOD, Mn-SOD, and GSH-Px was upregulated, while the expression of COX-2, iNOS, and IκB-α was downregulated by the polyphenols of PT and DHT. Thus, PTP and DHTP improved the antioxidant capacity of the liver. We identified the key regulatory genes of lipid metabolism in the liver to validate their regulatory effects on lipid metabolism. The researchers discovered that the polyphenols PT and DHT significantly reduced the gene expression of FAS, SREBP1-c, and C/EBP-α in the liver. PPAR-α, LDLR, and CPT-1a gene expression, on the other hand, increased. Therefore, PTP and DHTP enhanced the liver’s antioxidant capacity and regulated lipid metabolism.

In order to investigate the molecular mechanisms of the two tea polyphenols on antioxidation and lipid metabolism regulation in mice, the liver tissues of the H, R, and P groups were analyzed using transcriptomics. Both PTP and DHTP were found to regulate the proteasome and p53 signaling pathways. P53, a well-known tumor suppressor protein that is also known as the genome guardian, regulates the expression of a number of genes involved in cell cycle regulation, redox balance (the production of antioxidant enzymes), DNA replication and repair, apoptosis, and autophagy (Holley and St Clair 2009; Saleem et al. 2009, 2014). Various forms of oxidative stress result in the post-translational modification of p53, enabling it to regulate genes for beneficial results (Liu and Xu 2011; Smeenk et al. 2008). Wu et al. 2019 found that *Lactobacillus* downregulated the p53 gene expression. Hence, PTP probably regulated the p53 pathway because of *Lactobacillus*. Proteasomes are responsible for regulating oxidative stress and lipid levels. Under oxidative stress, an increase in oxidative protein causes cell death. In contrast, the proteasome recognizes and degrades mild oxidative protein in the cytoplasm, nucleus, and endoplasmic reticulum, thereby minimizing its cytotoxicity (Davies 2001). Simultaneously, cells can maintain intracellular lipid levels by degrading the ubiquitin proteasome system (UPS) to regulate the level of the ester biosynthesis enzyme (Stevenson et al. 2016). In addition, DHTP and PTP controlled the PPAR signaling pathway and steroid biosynthesis, respectively. Peroxisome proliferator-activated receptor (PPAR) proteins belong to the steroid hormone receptor superfamily. They combine with the retinoid X receptors to form heterodimers. These molecules regulate genes involved in lipid and glucose metabolism, adipocyte differentiation, fatty acid transport, and inflammation (Cabrero et al. 2002; Dubuquoy et al. 2002). Steroids inhibit cholesterol synthesis, regulating lipid metabolism; steroids can also be used to protect the liver (Dembitsky 2021). Different pathways are regulated by two types of tea polyphenols. However, the majority of these pathways are associated with lipid metabolism and antioxidation. This finding supports the ability of PTP and DHTP to regulate various antioxidation and lipid metabolism pathways.

Finally, this study aimed to investigate the biological activity and molecular mechanism of Pu-erh and Dian Hong tea polyphenols in mice using 16 S rRNA gene sequencing and hepatic transcriptome analysis. Because of the different degree of fermentation, there were significant differences in the composition structure and beneficial function of PTP and DHTP. PTP and DHTP both have the potential to regulate the antioxidant and lipid metabolism of the liver by influencing different intestinal microbiota and signaling pathways in the host. This study adds to our understanding of the health benefits of Pu-erh and Dian Hong tea polyphenols.

## Supplementary Information

**Additional file 1**: **Table S1**. Primers sequences for Real-Time Quantitative PCR. **Table S2**. Data preprocessing statistics and quality control. **Table S3**. Sample sequencing data quality summary.

## Data Availability

The raw sequence data reported in this paper have been deposited in the Genome Sequence Archive (Genomics, Proteomics & Bioinformatics 2017) at National Genomics Data Center (Nucleic Acids Res 2021), China National Center for Bioinformation/Beijing Institute of Genomics, Chinese Academy of Sciences, under accession number CRA009184 and CRA009210 that are publicly accessible at https://bigd.big.ac.cn/gsa.
