# Supplementary material for: Effects of Pu-erh and Dian Hong tea polyphenols on the gut-liver axis in mice
Source: AMB Express. 2023 Jun 2;13:53. doi: 10.1186/s13568-023-01565-4 (PMC10236074; doi:10.1186/s13568-023-01565-4)
Supplement: Supplementary file 1 — Additional file 1: Table S1. Primers sequences for Real-Time Quantitative PCR. Table S2. Data preprocessing statistics and quality control. Table S3. Sample sequencing data quality summary. [file 13568_2023_1565_MOESM1_ESM.pdf]

Article title: Effects of Pu-erh and Dian Hong tea polyphenols on the gut-liver axis in mice.

Journal name: AMB Express

Author names:

Ning Wang[1,a,] Chaohua Lan[1,a], Muhammad Aamer Mehmood[1,4], Manli He[3], Xiongjun Xiao[1], Linman Li[1], Dalong Liao[1], Kewei Xu[1], Shan Mo[1], Puyu Zhang[1], Xiaoli Zhou[1], Baoxiang Gu[1], Hui Zhu[1,*], Tao Wu[2,*]

[1]College of Bioengineering, Sichuan University of Science and Engineering, Zigong 643000, China

[2]School of Food and Biological Engineering, Xihua University, Chengdu, 610039, China

[3]Laboratory Animal Center, Southwest Medical University, Luzhou, Sichuan, China

[4]Department of Bioinformatics and Biotechnology, Government College University Faisalabad, Faisalabad, Pakistan

[a]These authors contributed equally

corresponding author:

Hui Zhu

zhuhuiwn@outlook.com (H Zhu, Ph.D.)

College of Bioengineering, Sichuan University of Science and Engineering, Zigong 643000, China

Tao Wu.

wutaobox@gmail.com (W Tao, Ph.D.)

School of Food and Biological Engineering, Xihua University, Chengdu, 610039, China

Additional file Table S1 Primers sequences for Real-Time Quantitative PCR

Gene	Forward Sequence	Reverse Sequence
Cu/Zn-SOD	5’-AACCAGTTGTGTTGTCAGGAC-3’	5’-CCACCATGTTTCTTAGAGTGAGG-3’
Mn-SOD	5’-CAGACCTGCCTTACGACTATGG-3’	5’-CTCGGTGGCGTTGAGATTGTT-3’
GSH-Px	5’-CCACCGTGTATGCCTTCTCC-3’	5’-AGAGAGACGCGACATTCTCAAT-3’
PPAR-α	5’-TCACACAATGCAATCCGTTT-3’	5’-GGCCTTGACCTTGTTCATGT-3’
LDLR	5’-CAGCTCTGTGTGAACCTGGA-3’	5’-TTCTTCAGGTTGGGGATCAG-3’
CPT-1a	5’-ATGACGGCTATGGTGTCTCC-3’	5’-GTGAGGCCAAACAAGGTGAT-3’
C/EBP-α	5’-GCCAAGAAGTCGGTGGATAA-3’	5’-CCTTGACCAAGGAGCTCTCA-3’
FAS	5’-GGGACACTCCACACCAGAGT-3’	5’-TAGACGTCAGCAGGTCGATG-3’
SREBP-1c	5’-GGCATGAAACCTGAAGTGGT-3’	5’-TGCAGGTCAGACACAGGAAG-3’
β-actin	5’-AGCCATGTACGTAGCCATCC-3’	5’-TTAAGCCATGCTCTGCAATG-3’

Additional file Table S2 Data preprocessing statistics and quality control

#Sample_name	Raw_reads(#)	Clean_Reads(#)	Base(nt)	AvgLen(nt)	Q20	GC%	Effective%
L_H1	80138	80138	32967865	411	78.26	52.93	100
L_H2	80115	80115	32835944	409	77.7	52.7	100
L_H3	80117	80117	33147169	413	78.89	52.9	100
L_H4	80065	80065	32881418	410	77.48	52.69	100
L_H5	80124	80124	33028899	412	76.48	52.57	100
L_H6	80213	80213	32910713	410	75.72	53.36	100
L_H7	80063	80063	32753434	409	77.72	52.94	100
L_H8	80176	80176	32923558	410	76.68	53.11	100
L_P1	88066	80284	32866220	409	84.54	52.52	91.16
L_P2	87578	80289	33209376	413	84.9	52.38	91.68
L_P3	82649	80042	32917100	411	85.23	52.32	96.85
L_P4	83681	80232	32914567	410	84.88	52.35	95.88
L_P5	83190	80075	32720333	408	84.74	52.41	96.26
L_P6	86588	80134	32931179	410	83.35	52.13	92.55
L_P7	88927	80060	33007306	412	85.27	52.18	90.03
L_P8	86597	80344	33184453	413	85.54	52.05	92.78
L_R1	80237	80237	33097154	412	79.13	52.94	100
L_R2	80092	80092	33111533	413	80.99	53.09	100
L_R3	80052	80052	32838845	410	79.66	53.1	100
L_R4	80096	80096	32887856	410	79.59	53.34	100
L_R5	80034	80034	33093350	413	80.52	52.82	100
L_R6	80140	80140	32999059	411	79.33	53.31	100
L_R7	80095	80095	32890101	410	79.66	53.41	100

L_R8	80067	80067	32901858	410	79.82	53.33	100

Note： Raw reads : Filter out the sequence of low quality bases; Clean reads: After filtering the chimeras, the final sequences are used for subsequent analysis; Base: The number of bases of the final Clean reads; AvgLen(nt) : The average length of Clean reads; Q20: Percentage of bases in Clean reads with base mass values greater than 20 (sequencing error rate less than 1%); GC (%): The content of GC bases in Clean reads; Effective (%): Number of Clean reads versus percentage of Raw reads.

Additional file Table S3 Sample sequencing data quality summary

sample	library	raw_reads	clean_reads	clean_bases	error_rate	Q20	Q30	GC_pct
L_H1	L_H1	136637760	131737204	9.88G	0.02	98.34	94.99	49.1
L_H2	L_H2	125289136	121093804	9.08G	0.02	98.15	94.52	49.5
L_H3	L_H3	130222476	126083536	9.46G	0.02	98.22	94.72	49.54
L_R1	L_R1	131669892	127200556	9.54G	0.02	98.27	94.69	49.78
L_R2	L_R2	151466880	146302280	10.97G	0.02	98.15	94.44	49.34
L_R3	L_R3	142025456	137049552	10.28G	0.02	98.36	94.95	49.19
L_P1	L_P1	149093104	142991464	10.72G	0.02	98.31	94.85	49.14
L_P2	L_P2	151589972	141192488	10.59G	0.02	98.34	94.96	49.28
L_P3	L_P3	150489480	143967984	10.8G	0.02	98.28	94.71	49.43

Note: raw_ Reads: the number of reads in the raw data, clean_ Reads: the number of filtered reads of the raw data, clean_ Bases: base number of filtered raw data (clean base = clean reads * 150bp), error_ Rate: overall sequencing error rate of data, Q20: percentage of bases with phred value greater than 20 in total bases, Q30: percentage of bases with phred value greater than 30 in total bases, GC_ PCT: percentage of G and C in four bases of clean reads.
